# Respiratory Support of Infants With Congenital Diaphragmatic Hernia

**DOI:** 10.3389/fped.2021.808317

**Published:** 2021-12-24

**Authors:** Emma Williams, Anne Greenough

**Affiliations:** ^1^Department of Women and Children's Health, School of Life Course Sciences, Faculty of Life Sciences and Medicine, King's College London, London, United Kingdom; ^2^Asthma UK Centre for Allergic Mechanisms in Asthma, King's College London, London, United Kingdom; ^3^National Institute for Health Research (NIHR) Biomedical Research Centre at Guy's and St Thomas' National Health Service (NHS) Foundation Trust and King's College London, London, United Kingdom

**Keywords:** mechanical ventilation, pressure controlled ventilation, volume controlled ventilation, high frequency oscillation, surfactant, inhaled nitric oxide

## Abstract

Optimisation of respiratory support of infants with congenital diaphragmatic hernia (CDH) is critical. Infants with CDH often have severe lung hypoplasia and abnormal development of their pulmonary vasculature, leading to ventilation perfusion mismatch. It is vital that lung protective ventilation strategies are employed during both initial stabilisation and post-surgical repair to avoid ventilator induced lung damage and oxygen toxicity to prevent further impairment to an already diminished gas-exchanging environment. There is a lack of robust evidence for the routine use of surfactant therapy during initial resuscitation of infants with CDH and thus administration cannot be recommended outside clinical trials. Additionally, inhaled nitric oxide has been shown to have no benefit in reducing the mortality rates of infants with CDH. Other therapeutic agents which beneficially act on pulmonary hypertension are currently being assessed in infants with CDH in randomised multicentre trials. The role of novel ventilatory modalities such as closed loop automated oxygen control, liquid ventilation and heliox therapy may offer promise for infants with CDH, but the benefits need to be determined in appropriately designed clinical trials.

## Introduction

The developmental disruption to the lungs and pulmonary vasculature of newborn infants with congenital diaphragmatic hernia (CDH) poses challenges during adaptation to postnatal life. The function of the lungs in providing essential gas exchange of oxygen and carbon dioxide at the alveolar-capillary membrane can be greatly diminished in infants presenting with this congenital abnormality (1). Importantly, appropriate, and timely intervention by clinicians is necessary to provide what can often be life-saving treatment, but may further adversely affect gas exchange. Ventilatory strategies and modalities are continuously being developed, with advances in technology contributing to such techniques. This review aims to provide clinicians with an outline of the recent evidence based ventilatory options available from birth and the initial stabilisation, through to surgery and post-operative management. It will also provide an insight into future developments.

## Ventilatory Management During Resuscitation

Guidelines from the European Congenital Diaphragmatic Hernia (CDH EURO) Consortium and the American Academy of Pediatrics and American Heart Association (AAP/AHA) recommend routine intubation at birth of all infants with CDH where the prenatal diagnosis is known, with avoidance of bag and mask ventilation and the resultant inflation of herniated bowel contents (2). Peak inspiratory pressures of <25 cmH_2_O are recommended to avoid ventilator induced lung injury (VILI) to both lungs (2).

Respiratory function monitoring can measure the response to initial resuscitation and the results utilised to predict subsequent survival. Expiratory tidal volumes (*p* = 0.009) and lung compliance (*p* = 0.03) measured during the first minute of recorded resuscitation have been shown to be lower in non-survivors. An expiratory tidal volume of >3.8 ml/kg and a lung compliance of >0.12 ml/cmH_2_0/kg was reported to be predictive of survival with 85% sensitivity and 90% specificity. Furthermore, tidal volumes of spontaneous breaths measured during the first 10 min after intubation have been described to be lower in those in those who either died prior to discharge or in those who developed chronic lung disease compared to survival without chronic lung disease (2.0 vs. 4.3 ml/kg; *p* = 0.004) (3). The achievement of higher maximal pre-ductal oxygen saturations (100 vs. 93%; *p* = 0.037) prior to transfer to neonatal intensive care have also been associated with greater survival in infants with a diagnosis of CDH (4). Such results are reflective of the degree of pulmonary hypoplasia in non-survivors. Additionally, respiratory function monitoring can be utilised to calculate the anatomical dead space in those with congenital malformations affecting the lungs. A larger anatomical dead space has been reported in those infants with CDH who survived to discharge [2.9 (2.8–3.3) ml/kg] compared to those who died [2.2 (2.1–2.7) ml/kg; *p* = 0.003] and can be used to predict survival [area under the curve (AUC) = 0.90] (5).

**Figure 1:**
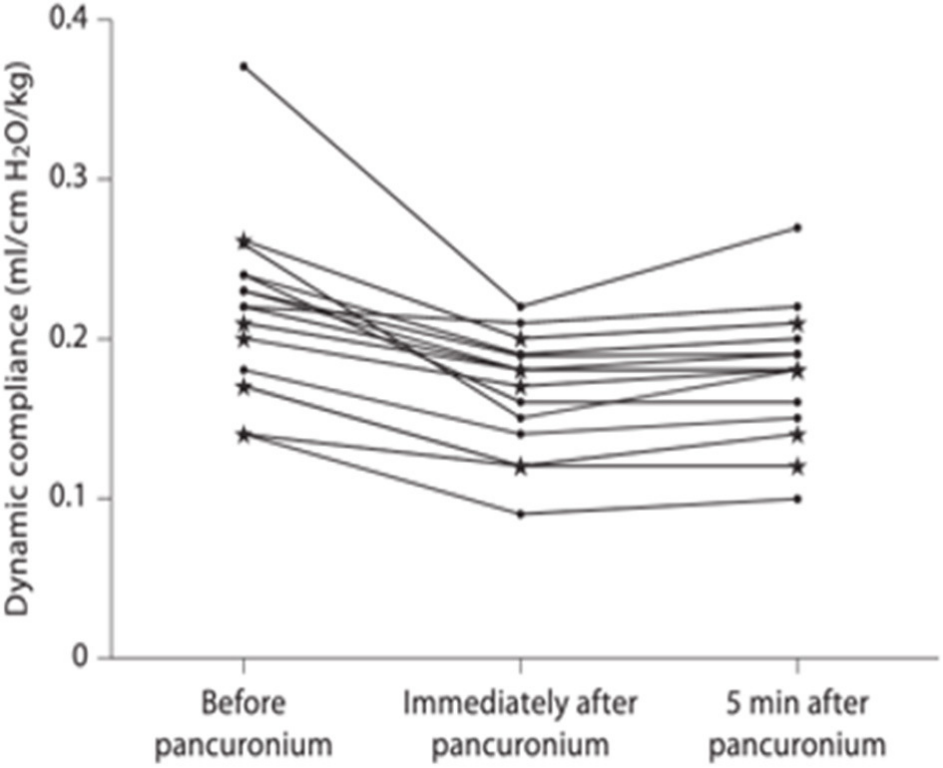
Dynamic compliance immediately before, immediately after and 5 min after pancuronium bromide administration [taken from (6)].

Dynamic lung compliance is low at birth and has been shown to be adversely affected by administration of a neuromuscular blocking agent. Indeed, the median lung compliance in a cohort of 15 infants with antenatally diagnosed CDH reduced from 0.22 to 0.16 ml.cmH_2_O^−1^.kg^−1^ (*p* < 0.001) immediately post administration of pancuronium bromide (6) (Figure 1). Hence, neuromuscular blocking agents should not be routinely administered during resuscitation (Figure 1). The respiratory function monitor used for pulmonary assessment within this study did not, however, measure oesophageal pressure

During resuscitation, physiological based cord clamping in animal models has been shown to be beneficial as dilatation of the pulmonary vasculature occurs following lung aeration, and thus pulmonary blood flow increases and oxygenation is improved (7). The feasibility of intact cord resuscitation in infants with CDH has recently been assessed in pilot studies. One prospective observational study (*n* = 40) reported no increase in neonatal adverse events whilst initiating resuscitation prior to cord clamping (8). A single-arm safety study (*n* = 20) determined the feasibility of intubation and ventilation prior to cord clamping and subsequently reported no significant difference in oxygenation indices or need for subsequent vasoactive therapy compared to those CDH infants undergoing immediate umbilical cord clamping (9). Recruitment is in progress into a multicentre international randomised trial determining the impact of physiological based cord clamping on clinically relevant outcomes in infants with CDH (10).

Where adequate antenatal development of the lung is expected, spontaneous breathing at birth can be considered. A recent, retrospective cohort study of 18 infants with mild CDH found a spontaneous breathing approach at delivery to be both safe and feasible (11). Prospective randomised trials are needed to appropriately evaluate this approach.

## Initial Ventilatory Management Pre-surgery

**Figure 2:**
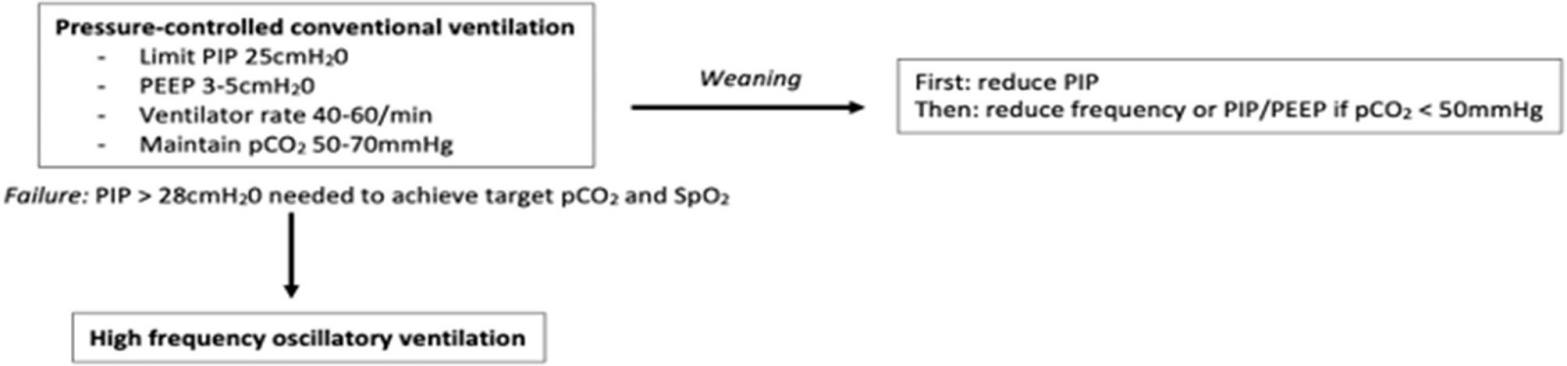
Flow diagram of initial ventilatory strategies.

Determining the optimal initial mode of ventilation in infants with CDH was assessed in a randomised trial (the VICI-trial) (12). Lung volume recruitment strategies utilised by high frequency oscillatory ventilation (HFOV) were not found to be superior to conventional mechanical ventilation (CMV) in reducing the combined outcome of death or bronchopulmonary dysplasia (BPD) [OR 0.62 (95% CI 0.25–1.55), *p* = 0.31]. With regards to secondary outcomes, however, the median (IQR) duration of mechanical ventilation was lower in the CMV group [10 (6–18) days] than the HFOV group [13 (8–23) days, *p* = 0.03]. Given the underlying lung pathology in CDH is that of pulmonary hypoplasia (a non-recruitable lung disease) this likely explains the inferiority of HFOV compared to CMV. Of those randomised to CMV, 42.9% required treatment with inhaled nitric oxide, compared to 56.2% of those on HFOV (*p* = 0.045). Additionally, a greater duration of vasoactive medication was needed in the HFOV [8 (4.3–19) days] compared to the CMV group [6 (3.3–11.8) days, *p* = 0.02]. The VICI trial also showed conventional ventilatory support to be beneficial in reducing the requirement for extracorporeal membrane oxygenation support (ECMO) (26 vs. 51%, *p* = 0.007) and is thus suggested as first line choice of ventilatory support in infants with CDH (2, 13, 14). Such benefits of CMV seen with regards to the secondary outcomes may however be reflective of the higher starting mean airway pressure (MAP) in the HFOV group (initial MAP 13–17 cmH_2_0). A recent, multicentre cohort study of 328 infants also demonstrated no significant differences between HFOV or CMV as initial mode of ventilation when reporting on the outcome of mortality [OR 0.98 (95% CI 0.57–1.67)] or BPD [OR 1.66 (95% CI 0.50–5.49)] (15). That study, however, is limited by its retrospective nature, although propensity score matching was performed to reduce potential confounding. Furthermore, no significant difference was reported between mode of respiratory support (HFOV or CMV) at the time of surgical repair when considering oxygen dependency or death at 28 days (16). Use of high positive end-expiratory pressure (PEEP) may cause lung injury by over-inflation of the ipsilateral lung during HFOV and the ensuing pulmonary inflammatory response (12, 17). Nevertheless, use of HFOV may be indicated as rescue therapy following failure of initial conventional ventilatory strategies (18). Failure of conventional ventilation in CDH infants is considered when peak inspiratory pressures higher than 28 cmH_2_O are required to maintain oxygen saturations within target range and the partial pressure of carbon dioxide (pCO_2_) between 50 and 70 mmHg (2) (Figure 2).

## Lung Protective Ventilatory Strategies

### Pressure-Controlled Ventilation and Permissive Hypercapnia

Pressure controlled ventilation and permissive hypercapnia are strategies employed to avoid damage to the lung contralateral to the herniation. Indeed, a reduction in mortality was demonstrated in infants with CDH when pCO_2_ levels as high as 70 mmHg were permitted (42.9 vs. 14.3%; *p* = 0.002) (19). That study, however, only reported outcomes at a single institution before and after the introduction of permissive hypercapnia as a therapeutic strategy over a 16-year period. The results may, therefore, have been influenced by other significant changes and advances in management during that time. To avoid ventilatory induced lung damage, low ventilatory pressures are advised by the CDH EURO Consortium, with peak inspiratory pressures of <25 cmH_2_O recommended for use if possible, however that upper limit is not evidence based (2, 20).

### Volume-Targeted Ventilation

The tidal volumes required to maintain effective minute ventilation and clearance of carbon dioxide in infants with CDH have been reported to be similar to that of control infants both pre (4.7 vs. 4.9 ml/kg; *p* = 0.49) and post (4.5 vs. 4.9 ml/kg; *p* = 0.14) operatively, however, in that retrospective cohort study, episodes in which tidal volumes corresponded with hypercapnic episodes were excluded. The most appropriate tidal volume targets in infants with CDH remains unanswered. Indeed, too low tidal volumes will result in an increase in the dead space to tidal volume ratio, yet use of too high tidal volumes may over-distend the already fewer alveoli existing in hypoplastic CDH lungs (20). No randomised trials have yet been performed to determine the optimal tidal volumes targets in infants with pulmonary hypoplasia secondary to congenital diaphragmatic hernia (21).

## Intra-operative Ventilation

A pilot, randomised controlled trial aimed to determine the effect of open or thorascopic repair on intraoperative acidosis and hypercapnia. The thorascopic approach was associated with higher levels of intraoperative partial pressure of carbon dioxide (pCO_2_) (83 vs. 61 mmHg; *p* = 0.036) and prolonged acidosis compared to open CDH repair (14). The insufflation of CO_2_ during thorascopic repair was thought to adversely impact upon intra-operative ventilation; all the infants were supported by conventional ventilation. Subsequent potential countermeasures to hypercapnia during thorascopic repair, such as intrapulmonary percussive ventilation and pausing the insufflation of CO_2_ during surgery, have resulted in non-significant differences in the maximal pCO_2_ levels during thorascopic [55.9 (38–192) mmHg] and laparoscopic repair [54.1 (41–72) mmHg] (*p* = 0.60) (22). An observational study reported fewer infants with CDH to have hypercapnic (>60 mmHg) or hypoxic (SpO_2_ < 90%) episodes during thorascopic assisted repair than open repair, with a shorter duration of post-operative mechanical ventilation in the former group (*p* < 0.05) (23), but there was no reported significant difference in the overall post-operative survival rates between either choice of surgical repair.

Retrospective cohort analysis of infants who underwent thorascopic repair reported an increase in intraoperative pCO_2_, with subsequent acidosis regardless of whether conventional or high frequency ventilation was used. Intra-operative pCO_2_ was not significantly different when HFOV or CMV were chosen as the ventilatory modality during surgery; however, the infants receiving HFOV during thorascopic repair exhibited less marked respiratory acidosis compared to their pre-surgery values than infants supported by conventional ventilation (24). These results suggest that utilisation of HFOV during thorascopic repair may prevent deterioration of respiratory acidosis to a larger degree than conventional ventilation.

## Ventilatory Management Post-surgical Repair

Respiratory compliance has been reported to decrease in infants with CDH following surgical repair (25). Furthermore, weight corrected respiratory system compliance (Crs) measured following surgery, in those infants with left sided CDH, has been shown to be negatively correlated with the need for prolonged post-operative mechanical ventilation (*p* = 0.006) (26). Positive end-expiratory pressure (pEEP) levels following surgical repair can affect respiratory system compliance and resistance in those with mild-moderate CDH and persistent pulmonary hypertension. In a randomised 1-h crossover trial, lung compliance increased by 30% when PEEP levels of 2 cmH_2_O were applied in comparison to use of 5 cmH_2_O of PEEP (*p* = 0.0001) (27). The improvement in oxygenation that occurred at the lower level of PEEP of 2 cmH_2_O, suggests higher PEEP levels are associated with over-distension of aerated “open” alveoli primarily within the ipsilateral lung (27). That study, however, was performed after surgical repair of the defect and so it remains to be answered whether low PEEP levels are beneficial pre-repair of the defect, given that the presence of viscera within the chest cavity may indeed prevent over-distension of the ipsilateral lung. Furthermore, high PEEP levels immediately after birth have been shown in an animal model to be beneficial in establishing functional residual capacity (28).

Prone positioning of mechanically ventilated infants post-operative repair of CDH has been associated with an improvement in oxygenation (PaO_2_/FiO_2_ ratio) (*p* = 0.032) and a reduction in the alveolar-arterial oxygen difference (*p* = 0.043) (29). Limitations of that study, however, were that measures of oxygenation and respiratory function were only measured for 30 min in each position and were not related to the adverse longer-term pulmonary outcomes suffered by infants with CDH. Post-surgical ventilation with tidal volumes of <5 ml/kg have been associated with an increase in the work of breathing in infants with CDH (*p* = 0.001) (30). That study, however, only included seven infants and future randomised, adequately powered studies are necessary to determine optimal tidal volume levels pre and post repair. Furthermore, the impact of such ventilatory strategies in relation to long-term pulmonary outcomes needs to be ascertained.

## Additional Therapies

### Supplementary Oxygen

There is a lack of randomised control trials determining the optimal fraction of supplemental oxygen during resuscitation in infants with CDH. In view of such paucity of robust data it is speculated that a starting fraction of the inspired oxygen (FiO_2_) of <1.0 during the initial resuscitation of newborn infants with CDH may be beneficial, with subsequent titration of the FiO_2_ to maintain preductal peripheral oxygen saturations (SpO_2_) between 80 and 95% (2). This starting FiO_2_ comes as a consequence of increasing concern related to the unfavourable effects of oxidative stress (31). Reducing free radical formation by lowering the levels of the inspired oxygen may subsequently reduce pulmonary vasoconstriction and the associated adverse consequences (32–34). Furthermore, animal models of persistent pulmonary hypertension of the newborn (PPHN) have shown that resuscitation with high levels of inspired oxygen (FiO_2_ 1.0 vs. 0.5) can also impair the later pulmonary vasodilator response to inhaled nitric oxygen (iNO) which may be required as rescue therapy for respiratory failure in infants with CDH (35, 36). A lower starting FiO_2_ (0.5) during the resuscitation of infants with CDH has been shown to have no adverse effects upon survival (adjusted *p* = 0.142) or need for ECMO (adjusted *p* = 0.159) than starting resuscitation at a higher FiO_2_ (1.0) (37). If the SpO_2_, however, remained low and there was a subsequent need to increase the FiO_2_ to 1.0, then this was associated with a trend in reduced survival rates and postnatal ECMO requirement, but the worse outcomes were no longer significant after controlling for a lower gestational age at birth, liver position and lung-head ratio (LHR) (37). One limitation of those results is the retrospective cohort nature of the study. Future trials that randomise newborn infants with CDH to different starting levels of FiO_2_ during resuscitation are needed to provide clinicians with evidence based FiO_2_ targets and the later relationship to postnatal outcomes.

Knowledge of the partial pressure of arterial oxygen (PaO_2_) values are necessary to guide oxygenation indices and determine criteria for ECMO, with continuous monitoring of SpO_2_ utilised to guide optimal ventilatory strategies. The aim stated by consensus guidelines, is to achieve preductal oxygen saturations of between 80 and 95% 2 h after birth, with post ductal saturations above 70% (2). Provision of supplemental oxygen titrated to such SpO_2_ levels does, however, need careful monitoring (38, 39). Animal models of the CDH ventilatory responses during the first 2 h after birth have recently demonstrated unintentional cerebral hyperoxia to occur when cerebral blood flow is unmonitored, thought to be related to the rapid increases in carotid blood flow (38). Cerebral oxygenation, monitored by near-infrared spectroscopy, has been reported to be reduced during surgical repair of infants with severe CDH, regardless of conventional or high frequency modes of ventilatory support (*p* = 0.0001) (39). Those infants receiving HFOV however exhibited a prolonged reduction in cerebral oxygenation and required a longer duration of time to recover to normal values than those in the conventional group (*p* = 0.003) (39). The study, however, was not appropriately powered to determine the effects of mode of ventilation on cerebral oxygenation during surgery. Furthermore, the relationship of such findings with longer term neurodevelopmental outcomes were not reported.

### Use of Inhaled Nitric Oxide

Inhaled nitric oxide (iNO) use in newborn infants with CDH has been reported in 68 (97.1%) participating centres in the Congenital Diaphragmatic Hernia Study Group registry (40). Of 2,174 infants diagnosed by echocardiography with pulmonary hypertension (PH) 74.2% received iNO therapy, however 36.4% of infants without PH were also treated with iNO. Propensity score analysis revealed iNO use to be associated with a 15% higher absolute mortality rate (average treatment effect on the treated: 0.15; 95% CI 0.10–0.20) (40). The use of iNO therapy for CDH infants with hypoxemic respiratory failure, unresponsive to conventional therapy, has also not been found to decrease the need for ECMO or reduce the primary outcome of death before discharge (41). Furthermore, only 16% of infants in that study (41) fully responded to iNO therapy at a dosage of 20 parts per million (ppm), as determined by an improvement of oxygenation indices. Inhaled nitric oxide is, however, often the first line drug of choice for pulmonary hypertension in newborn infants with CDH and forms a standard of care for the CDH Euro Consortium group (2). Nevertheless, as iNO has not been shown to be beneficial in reducing mortality rates, it is not routinely recommended by the American Pediatric Surgical Association (APSA) for the treatment of pulmonary hypertension in infants with CDH (13). Further trials are underway to determine if other therapeutic agents which act to reduce PH may offer more promise in infants with CDH. Currently, an international multicentre, randomised controlled trial is in the recruitment phase to assess whether intravenous sildenafil may be superior to iNO in reducing mortality in newborns with CDH (42). Additional trials are also setting out to determine the beneficial effects of inotropic agents, such as milrinone, in treating pulmonary hypertension caused by right and left ventricular dysfunction in CDH. Such therapy may improve left ventricular diastolic and systolic function, reduce afterload and subsequently lead to improved oxygenation in those with CDH. A randomised pilot trial is being conducted to determine the safety and feasibility of undertaking a larger multicentre trial (43).

### Surfactant Therapy

Infants with CDH often exhibit reduced lung compliance (44). The use, therefore, of postnatal surfactant has been considered in infants with CDH. Use of postnatal surfactant administered to term infants (>37 weeks' gestational age, *n* = 522) was reported to have no beneficial impact upon survival, nor did such treatment reduce the incidence of chronic lung disease or the need for extra-corporeal membrane oxygenation (ECMO) (45). Furthermore, retrospective data from the CDH registry reported that administration of surfactant to preterm infants (<37 weeks' gestation, *n* = 424) was associated with a greater risk of death before discharge [odds ratio (OR) 2.17, 95% CI: 1.5–3.2; *p* < 0.01] (46). Additionally, surfactant replacement given to infants >35 weeks of gestation whilst on ECMO was reported to have no beneficial effects on the outcomes of survival to discharge (OR 1.0, 95% CI 0.67–1.62; *p* = 0.87) or requirement for supplemental home oxygen (OR 1.04, 95% CI 0.6–1.8; *p* = 0.90) (47). Surfactant concentrations in human foetuses with CDH have been reported to be similar to those of age matched controls, moreover the maturation and storage of surfactant appears not adversely impacted by this congenital pulmonary abnormality (48). Routine surfactant administration to infants with CDH is therefore not currently recommended in the EURO consensus guidance (2). Randomised clinical trials (RCTs) for surfactant therapy in preterm infants with CDH are warranted. If postnatal surfactant is given it should be noted that the standardised dosage regimens for weight are likely to be inaccurate due to the degree of pulmonary hypoplasia (48).

## Novel Ventilatory Modalities

### Neurally Adjusted Ventilatory Assist

Neurally adjusted ventilatory assist (NAVA) may confer protection to hypoplastic lungs. During NAVA, ventilatory support is delivered in response to diaphragmatic electrical activity. The utility of NAVA in newborn infants with structural diaphragmatic abnormalities has not been widely studied and since this ventilatory mode is dependent upon detection of neural diaphragmatic signals certain challenges may arise. In one study, after primary repair, infants with CDH placed on invasive NAVA were shown to have no differences in peak or resting electrical activity of the diaphragm during respiration compared to control infants with no underlying diaphragmatic abnormality, nor did they require higher levels of NAVA support (49). A recent case control study (*n* = 16) found no significant difference in the NAVA level (*p* = 0.286) used post-surgical repair in infants with CDH compared to those without CDH of similar age and weight at the time of study. Furthermore, NAVA use in those with diaphragmatic hernia was associated with a reduction in ventilatory requirements and the need for sedative therapy (49). Two retrospective feasibility studies performed in infants with CDH following surgical repair showed that post-operative weaning of ventilation with both invasive (*n* = 10) and non-invasive (*n* = 7) NAVA to be successful (50, 51). Additionally, the short-term outcomes of infants with CDH who have been placed on invasive NAVA post-surgery were assessed and NAVA was associated with a decrease in mean airway pressure (*p* < 0.001) and respiratory severity score (*p* < 0.001) at 72 h post initiation (*p* < 0.001) (49).

### Closed Loop Automated Oxygen Control

Closed loop automated oxygen control systems have yet to be trialled in infants with CDH, but recent developments have shown promising results in optimising ventilatory support in other pulmonary conditions (52–56). Whether such a modality has a role in infants with congenital diaphragmatic anomalies has yet to be determined.

### Heliox Therapy

Utilising heliox as an adjunctive therapy to the ventilation of infants with CDH was shown in one retrospective cohort study to be beneficial in reducing levels of hypercapnia (68 vs. 49 mmHg; *p* < 0.001) and levels of maximal ventilatory support required from high frequency oscillatory ventilation, and thus may be one such therapy to improve gas exchange in those with lung hypoplasia (57). Prospective randomised trials are required to ascertain the benefit of such therapy on short- and long-term pulmonary outcomes.

### Liquid Ventilation

Lung growth *in utero* occurs as pulmonary fluid secretion provides a continuous distending pressure to the airways (58). Fluid filled lungs, combined with foetal breathing movements, underlie the mechanisms behind antenatal lung growth (59). Liquid at room temperature and less viscous that water, perfluorocarbons may thus provide some benefit when conventional ventilatory strategies remain challenging during postnatal life (60). By providing constant distending pressure, liquid ventilation may improve lung mechanics in a similar fashion to that of an increased PEEP. Results from animal models of severe respiratory failure have indeed shown partial liquid ventilation to be beneficial with regard to gas exchange and reducing pulmonary shunting (88 vs. 31%; *p* < 0.001) during ECMO (61). A randomised trial to assess the feasibility of partial liquid ventilation (perfluorocarbon-induced lung growth) in newborn infants with CDH (*n* = 13) confirmed the short-term safety of performance of this novel technique during ECMO (62). Larger randomised trials would be required to ascertain the long-term safety and benefits of liquid ventilation as a postnatal strategy in infants born with congenital diaphragmatic hernia, however as obtaining regulatory approval may prove challenging such therapy may be of doubtful clinical relevance.

## Long Term Lung Function

Pulmonary morbidity and long-term lung function of infants with CDH is now of more significance as survival rates have increased (63). Follow up of 28 children with repaired left sided CDH (mean age 6.2 years) revealed 25% had abnormal pulmonary function (*p* < 0.01). Furthermore, those with abnormal pulmonary function had lower total lung volume on structural evaluation of chest tomography (826.5 ± 133.6 mls) than those with normal overall lung function (1,244.5 ± 407.9 mls; *p* < 0.05) (64). Longitudinal analysis of ventilation-perfusion (V/Q) mismatching has been recently reported in CDH survivors (65). In those with severe disease ipsilateral V/Q mismatch worsenend over time likely due to the progressive reduction in pulmonary perfusion (*p* = 0.012). Such perfusion deficits may be related to abnormal lung function and V/Q studies may thus be an important addition to exercise testing in order to identify and monitor those most at risk of worse outcomes.

## Conclusion

This review has highlighted respiratory management techniques which could be utilised in resusciation and during the pre and post operative management stages in infants with CDH. Respiratory function monitoring is a useful tool to monitor pulmonary mechanics and the results may be utilised to predict subsequent survival. Conventional ventilatory modes are recommenced as initial respiratory support given the primary lung pathology of pulmonary hypoplasia. Further research and more randomised trials are, however, needed to provide clinicians with evidence based, optimal respiratory management strategies which have been shown to improve the long term pulmonary outcomes of infants with congenital diaphgramatic hernia.

## Author Contributions

AG and EW designed the review and contributed to its production. Both authors contributed to the article and approved the submitted version.

## Funding

EW was supported by a grant from the Charles Wolfson Charitable Trust and a non-conditional educational grant from SLE. This research was supported by the National Institute for Health Research (NIHR) Biomedical Research Centre at Guy's and St Thomas' NHS Foundation Trust and King's College London.

## Author Disclaimer

The views expressed are those of the authors and not necessarily those of the NHS, the NIHR or the Department of Health.

## Conflict of Interest

The authors declare that the research was conducted in the absence of any commercial or financial relationships that could be construed as a potential conflict of interest.

## Publisher's Note

All claims expressed in this article are solely those of the authors and do not necessarily represent those of their affiliated organizations, or those of the publisher, the editors and the reviewers. Any product that may be evaluated in this article, or claim that may be made by its manufacturer, is not guaranteed or endorsed by the publisher.
